# Dual-Plane Midface Lift Through Transoral and Transtemporal Approach

**DOI:** 10.1007/s00266-024-04222-8

**Published:** 2024-07-15

**Authors:** Mehmet Gencer, Metin Kerem, Yunus Sağlam, Burak Ergün Tatar

**Affiliations:** 1 Mehmet Gencer Private Clinic, Plastic Surgery, Valikonağı street, Istanbul, Turkey; 2 Plastic Surgery Center, Istanbul, Turkey; 3 Department of Plastic, Reconstructive and Aesthetic Surgery, Rize State Hospital, Rize, Turkey; 4 Department of Plastic, Reconstructive and Aesthetic Surgery, Erzurum State Hospital, Erzurum, Turkey

**Keywords:** Midface lift, Transtemporal approach, Transoral approach, Dual-plane technique, Facial rejuvenation

## Abstract

**Introduction:**

The surge in the popularity of midface and temporal lifting procedures can be attributed to evolving social media trends and heightened patient expectations, particularly among younger individuals seeking “beautification” rather than traditional rejuvenation. Scarless techniques, such as transtemporal/transoral approaches, are increasingly preferred. This study aimed to combine the advantages of both superficial and deep dissection planes in midface and temporal lifting procedures.

**Methods:**

This retrospective study included 184 patients who underwent surgery using a dual-plane midface and temporal lift technique. Preoperative and postoperative assessments, including P1/P2 ratio calculations, were performed to evaluate volumetric distribution in the midface.

**Results:**

The study cohort exhibited a significant improvement in the P1/P2 ratio postoperatively (*p* < 0.05), indicating successful volume redistribution. Complications, including hypoesthesia, bruising, and infection, were managed effectively. Minor asymmetries were observed, with revisions offered, but declined by the patient.

**Discussion:**

This technique offers hidden incisions and reduces the risk of scar-related complications, making it suitable for patients seeking facial enhancement. Addressing the tear trough area and the lateral canthus provides comprehensive facial rejuvenation. The dual-plane approach facilitates both skin mobilization and volume shift, yielding favorable aesthetic outcomes.

**Conclusions:**

The dual-plane midface and temporal lift technique presented in this study offers a bi-vectoral approach to midfacial lifting, suitable for both beautification and rejuvenation goals. Despite the potential limitations, including infection risk, this method is an effective option for facial enhancement.

**Level of Evidence II:**

This journal requires that authors assign a level of evidence to each article. For a full description of these Evidence-Based Medicine ratings, please refer to the Table of Contents or the online Instructions to Authors www.springer.com/00266.

## Introduction

During the history of cosmetic surgery, midface and temporal lifting procedures have never been as popular as they are now, probably because of the social media culture [1]. Patient expectations are also getting higher over time, where lifted temples and brows, together with a fuller anterior upper cheek and slimmer lower cheek are essential [2]. Unlike before, these relatively younger patients target “beautification” rather than “rejuvenation” or erasing age-related symptoms [2–4]. Moreover, these highly demanding patients are seeking scarless procedures, which makes transtemporal/transoral approaches to the temple and midface areas more preferable than the transpalpebral approach. [5]

Temporal lifts and mid face lifts are often done together, and numerous techniques that simultaneously address both areas have been published earlier. [1, 6–8] Among the current techniques, some employ a deeper (sub-periosteal) dissection plane, whereas others are relatively superficial (sub-SMAS or subcutaneous). Both planes have their advantages and disadvantages.

In this study, we presented our cases of dual-plane midface lift through transoral and transtemporal approach.

## Materials and Methods

### Study Design

**Fig. 1:**
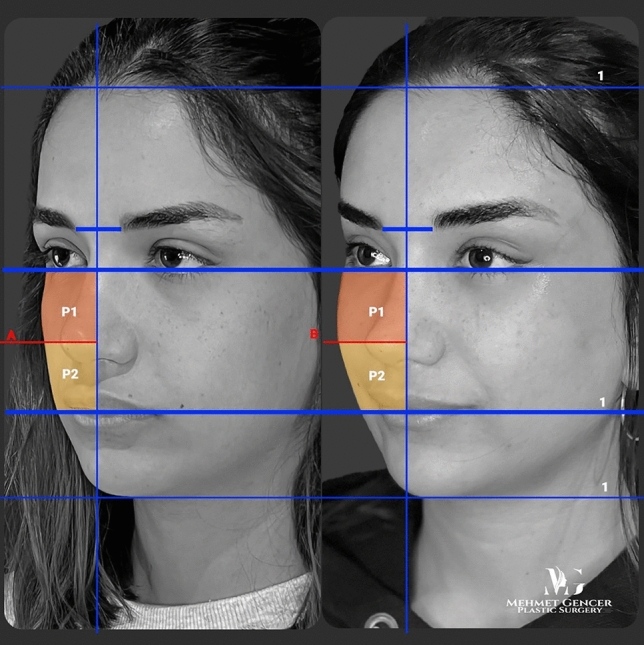
A vertical line passing through the midpoint of the medial aspect of the two eyebrows in the photographs. Subsequently, horizontal lines were drawn at the level of the lower eyelid margin and oral commissure. Thus, the midface area is bounded by two horizontal lines and one vertical line. Another horizontal line was drawn in the middle of these two horizontal lines, dividing the midface area into upper (P1) and lower parts (P2)

This retrospective study included 184 patients who underwent surgery using the dual-plane midface and temporal lift technique described by the senior author (M.G.) between 2021 and 2023. Written informed consent was obtained from all patients. Patients who underwent revision surgery or who had any additional medical conditions were excluded from the study. Preoperative and postoperative photographs were taken at the same level as the face, and at a distance of 2 m. A vertical line passing through the midpoint of the medial aspect of the two eyebrows was drawn in the photographs. Subsequently, horizontal lines were drawn at the level of the lower eyelid margin and oral commissure. Thus, the midface area is bounded by two horizontal lines and one vertical line. Another horizontal line was drawn in the middle of these two horizontal lines, dividing the midface area into the upper (P1) and lower parts (P2) (Fig. 1). P1/P2 ratio was then calculated using Adobe Photoshop CC24 software. The volumetric distribution in the midface, which was affected by the surgery, was evaluated using this ratio.

### Preoperative Evaluation

**Fig. 2:**
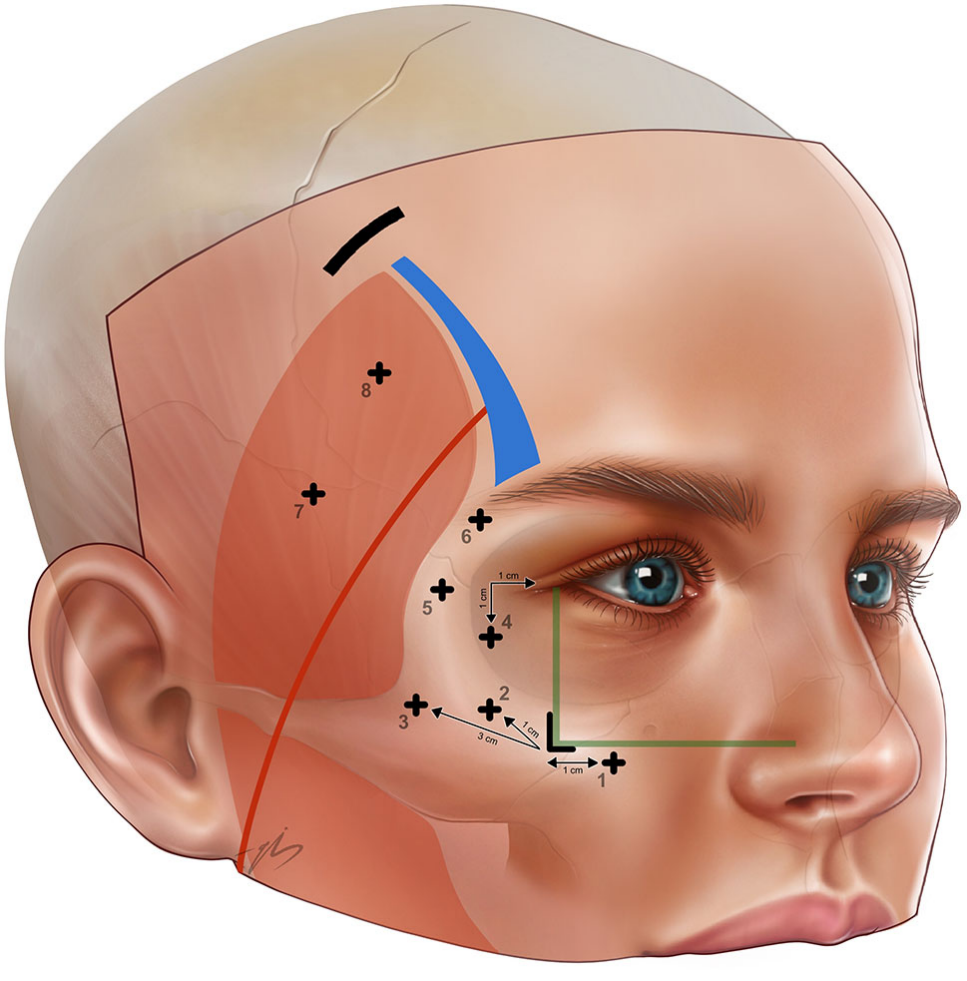
Surgical markings: oblique black line on the temporal region is the temporal incision, green lines are the vertical line drawn from the lateral canthus, and the horizontal line drawn at the level of the upper alar margin. Point 1 is 1 cm medial to the intersection point of two perpendicular green lines, point 2 is 1 cm superolateral to point 1. Point 3 was 2 cm superolateral to the 2. Point 4 was located 1 cm inferolateral to the lateral canthus. Point 5 was the lateral corner of the brow, and point 6 was 1 cm inferolateral to point 5 along the arc of the orbicularis muscle. The red line is the pitanguy line. Point 7 was 2 cm superior to the zygomatic arch and 1 cm posterior to the Pitanguy’s line. Finally, point 8 is 1, 5 cm superior to point 7

Markings were performed while the patient was sitting in an upright position. First, the vertical midline was marked in the frontal area. Subsequently, traces of the zygomatic arch and frontal branch of the facial nerve (pitanguy line) are depicted. The superior temporal septum was marked all the way from the upper rim into the hairline. A 2, 5 cm long incision line is marked 2 cm behind the temporal hairline, which perpendicularly intersects with the superior temporal septum. (Fig. 2) The lateral 1, 5 cm of the incision line was lateral, and the medial 1 cm of it is medial to the superior temporal septum. Thus, the incision line is generally parallel to the temporal hairline and almost perpendicular to the temporal crest.

The midface suspension points were marked after the temporal markings. Point 1 is 1 cm medial to the intersection point of two perpendicular lines, which are the vertical line drawn from the lateral canthus, and the horizontal line drawn at the level of the upper alar margin. (Fig. 2) Point 2 was 1 cm supero-lateral to point1. Point 3 was 2 cm superolateral to the 2. Point 4 was located 1 cm inferolateral to the lateral canthus. (Fig. 2) Point 5 is the lateral corner of the brow, and point 6 is 1 cm inferolateral to point 5 along the arc of the orbicularis muscle. To ensure that the frontal branch is safe, no suspension points were plotted in the zone between 0 and 5 cm. superior and 0, 5 cm inferior to Pitanguy’s line. If the points coincide with this zone due to the anatomy of the patient, they need to be modified and taken out of this “red zone.”

Point 7 was 2 cm superior to the zygomatic arch and 1 cm posterior to the Pitanguy’s line. Finally, point 8 is 1, 5 cm superior to point 7 (Fig. 2). Depending on the forehead width, hairline position, and periorbital symmetry issues, these points can be modified accordingly.

### Surgical Technique

All operations have been performed by the senior author (M.G.) under general anesthesia. All patients are asked to do a chlorhexidine mouthwash prior to surgery. The endotracheal tube is fixed to the labial frenulum and lingual frenulum. The space around the tube is carefully packed with a Miculitz gauze, avoiding fluid leakage to oropharynx during surgery. All the surgical field, including the oral cavity, is sterilized with povidone–iodine solution, and draped. 50 cc of 1/1.000.000 adrenaline solution is injected into the entire surgical site per side and waited for 15 min for the hemostatic effect to settle.

**Fig. 3:**
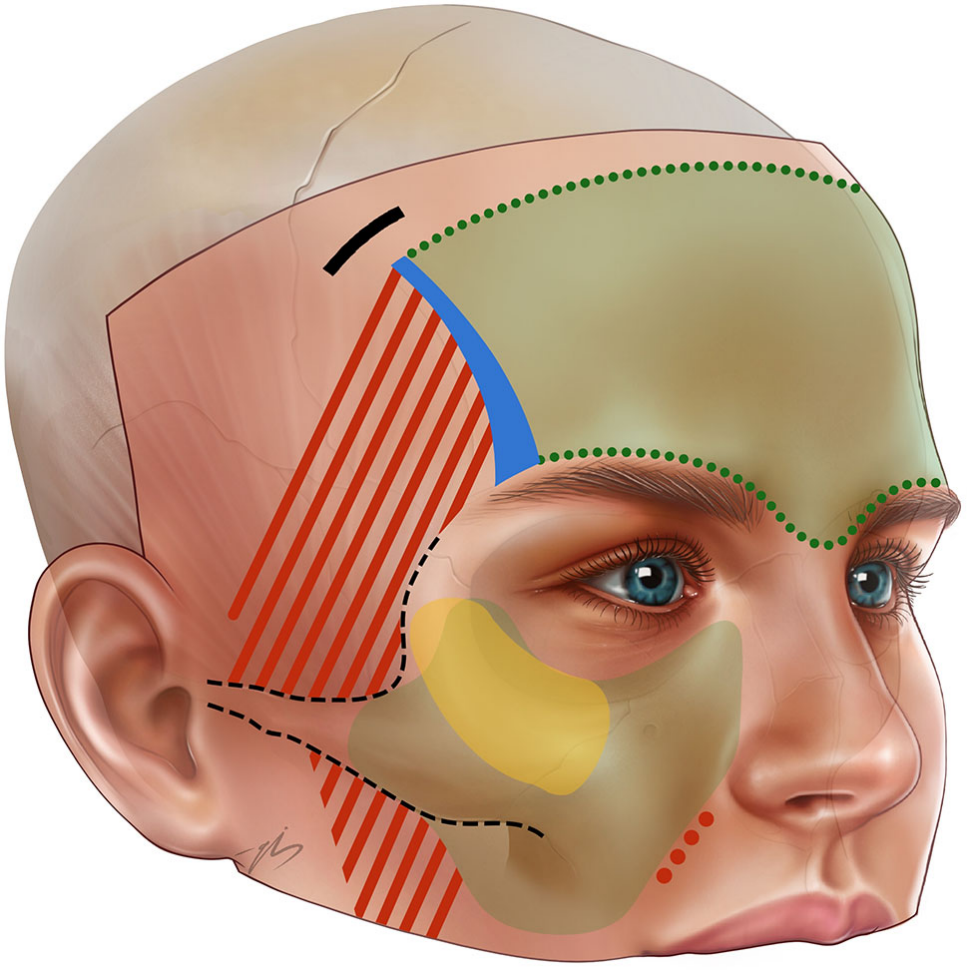
Temporal area incision; the green area shows subperiosteal dissection area, the red area shows subfascial dissection area and the yellow area shows dual-plane area

A 2.5 cm long incision, which was marked and explained previously, was made parallel to the hair follicles. Through this incision, the deep layer of the deep temporal fascia reached laterally, and the subperiosteal plane reached medial to the superior temporal septum. (Fig. 3) The superior temporal septum was bluntly dissected, and the two dissection planes lateral and medial to the STS were united. Subperiosteal dissection was performed over the entire frontal bone, taking care not to injure the supraorbital and supratrochlear nerves. To distribute the excess skin that will accumulate below the incision, the subperiosteal dissection is extended behind the incision line for 2 more centimeters. In the temporal area, dissection continued at the same level, down to 1 cm superior to the zygomatic arch. The inferior temporal septum, temporal ligamentous adhesions, and lateral brow thickening were removed. At the level of the superolateral orbital rim, the periosteum is incised with a semi-blunt elevator, and the rest of the dissection is performed subperiosteally afterward, down to the level of the lateral canthus. Here, the lateral orbital thickening was released, and the subperiosteal dissection was extended laterally over the zygomatic arch until Pitanguy’s line. All perforating vessels within the dissection field were cauterized near the deep surface (base) of the dissected space. Once this dissection was complete, all planes were checked for any persistent adhesions or intact ligaments. If so, any residual adhesion was freed.

The next, a superior gingivobuccal sulcus incision was made1 cm above the first premolar. This incision is performed deep, all the way down to the bone, giving access to the maxillary subperiosteal plane. In this plane, blunt dissection was continued laterally and superiorly. When the infraorbital bundle is seen, it is circumferentially dissected and gentle traction is applied to allow flap mobility. Medially, the dissection reached the nasomaxillary junction, ensuring that there was no adherence left medial to the infraorbital nerve. Inferolaterally, 1/3 of the medial portion of the tendinous attachment of the masseter to the zygomatic arch was included in the dissection zone. Dissection was performed over the masseter tendon, but the tendon was left intact with a bony origin. The dissection was extended 3 mm into the orbital cavity along the entire lower orbital rim, completely freeing the arcus marginalis. Superolateral medial portion of the zygomatic arch, which had already been dissected transtemporally, was checked again through the transoral approach.

**Fig. 4:**
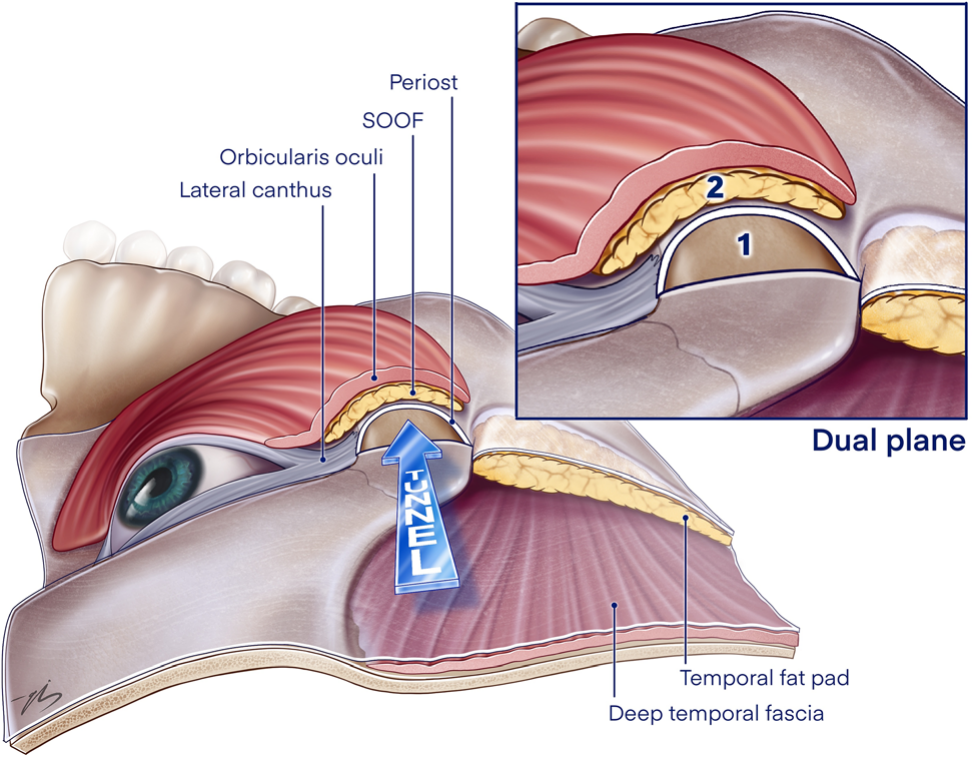
Tunnel at the level of the lateral orbital rim between the transtemporal and transoral dissection spaces. Area 1: lateral orbital wall bone on its floor, periosteum on its roof, area 2 has periosteum on its floor, orbicularis muscle on its roof, and SOOF inside

When transoral dissection is complete, the transtemporal and transoral dissection spaces are easily combined by creating a tunnel at the level of the lateral orbital rim. (Fig. 4) This is done using the temporal approach with a blunt dissector and direct finger pressure under direct vision. This tunnel was then widened to 2 cm by a Trépsat dissector. The roof of the unified plane is covered with the periosteum inferiorly and the orbicularis muscle, which is the extension of the SMAS at this level (1, 5). Right at the junction of these inferior and superior levels, a vertical blunt dissection was performed into the flap to access the SOOF and release it, approximately 2 cm caudally. This plane is dissected to obtain a softer transition between the subperiosteal and temporal dissection planes and is the superficial plane of the “dual plane” (sub-SMAS and sub-periosteal) described in this study. (Fig. 4)

**Fig. 5:**
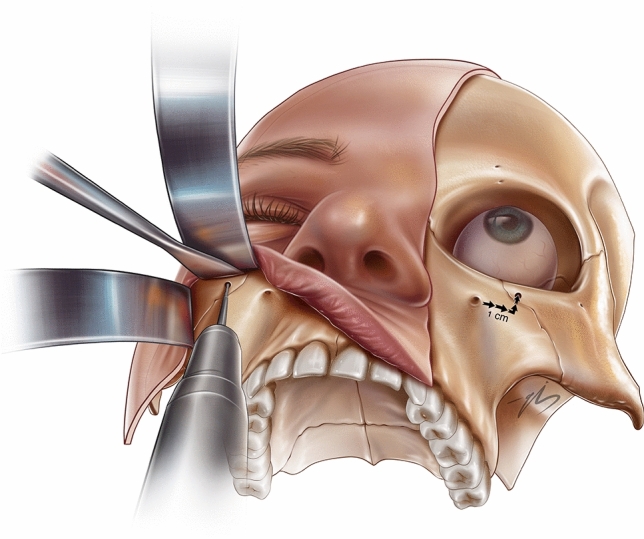
Left half: Via the trans-oral approach, a 2 mm bone tunnel is drilled at the lower orbital rim, 1 cm superolateral to the infraorbital nerve, using a micromotor drill. Rigft half: A bone tunnel placed 1 cm superolateral to the infraorbital nerve

**Fig. 6:**
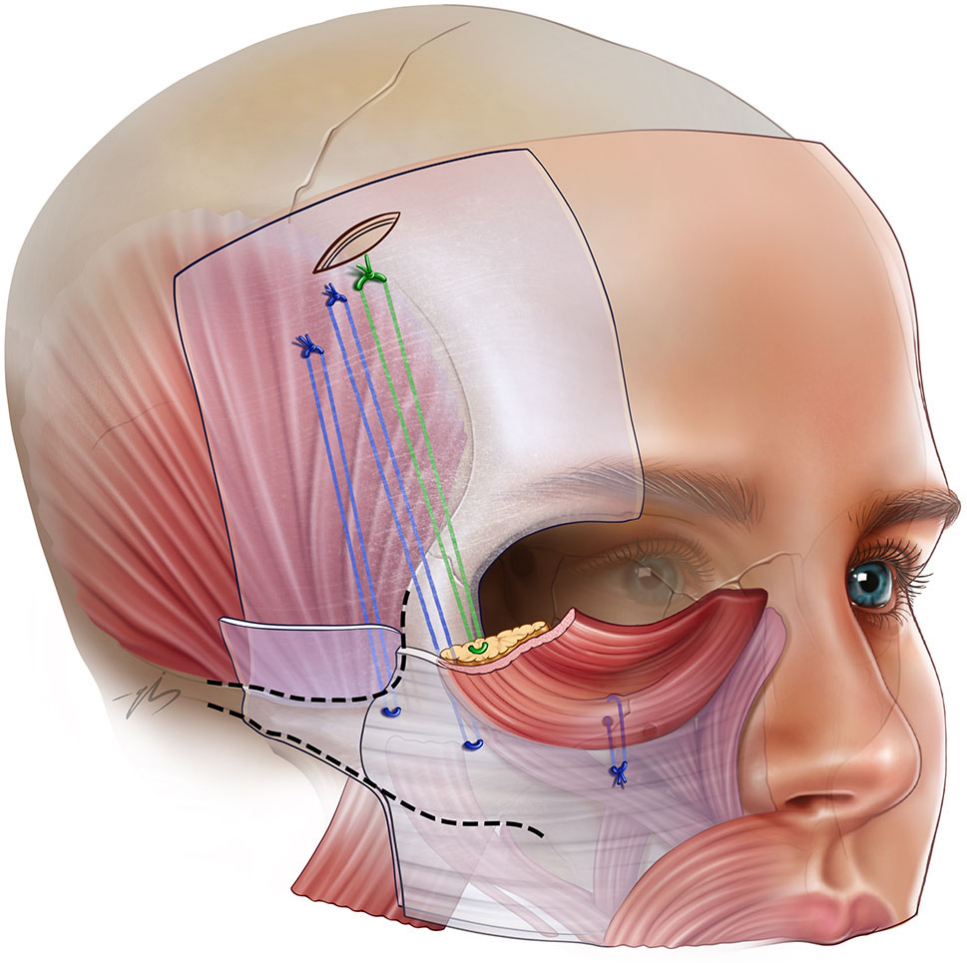
Fixation of areas 1-2-3-4, fixation of area 1 to the bone with blue suture, fixation of blue sutures 2 and 3 to the temporal area, green suture: fixation of SOOF point number 4 to the temporal area

After completing the dissection, the unified surgical plane was controlled both trans-temporally and trans-orally, ensuring that complete release was performed without any adherence left behind. via trans-oral approach, a 2 mm bone tunnel is drilled at the lower orbital rim, 1 cm superolateral to the infra-orbital nerve, by a micro-motor drill (Elec II mini micromotor (ELM-B405) (Fig. 5). The free end of a 2-0 prolene suture was passed through the bone tunnel, held with a clamp, and removed again to avoid needle contact with the orbital content. Using percutaneous guide needles, points 1, 2, and 3 were marked to clearly reflect the points on the deep surface of the midface flap. Then, at the level of points 2 and 3, 2/0 PDS sutures were passed through the deep surface of the flap, taking robust bites, including the periosteum and fascia. These two stitches were passed through a tunnel with a long needle holder introduced through the temporal incision. At point 1, a similar bite is taken from the flap by the prolene suture, the free end of which was initially passed through the infraorbital rim bony tunnel. First, a point 3 suture was fixed on the superior part of the DTF under moderate tension. Then, a point 2 suture was fixed on the DTF, 1 cm below where the point 3 suture was fixed **(**Fig 6**).** Finally, the point 1 suture is tied, fixing point 1 tightly to the rim. (Fig. 6) Once the midface fixation sutures are tied, one suture 1 cm lateral to the lateral canthus and one more suture 1 cm inferior to the former are passed through the SOOF at the plane defined as the superficial plane of the dual-plane structure (Fig. 6). These sutures were tightly tied to the relatively robust anchor area along the most cranial part of the temporalis muscle. In patients who required further canthal support (negative canthal tilt, congenital scleral show, iatrogenic ectropion), an optional 2/0 PDS static canthopexy suture was added. This optional stitch passes through lateral orbital thickening and is anchored to the origin of the temporalis muscle. All temporal areas, except the danger zone for the frontal branch of the facial nerve, were fixed to the origin of the temporalis muscle with four stitches. Two of them were along the marginal arc of the orbicularis muscle (points 5 and 6), and the other two were in the posterior zone. Once the fixation was bilaterally complete and everything was checked for symmetry, the temporal incisions were closed with 3/0 vicryl subcutaneous sutures and 4/0 monocryl skin closure. Intraoral incisions were closed using 5/0 vicryl rapide mattress sutures.

At the end of surgery, the malar area is tightly taped in a horizontal fashion, the temporal area diagonally, and the frontal area vertically, aiming to smoothen the skin folds and dimples. After taping, a headband was applied to obtain hemostatic compression.

### Postoperative Care

#### Hospital Follow-Up

Following the surgical procedure, patients are typically kept under observation at the hospital overnight.

#### Duration of Taping and Headband Use

Tapes are usually kept in place for a minimum of 7 days, while the headband is only recommended for the first day.

#### Ice Compression

Applying ice compression to the malar and periorbital areas for 10 min/h is advised for the initial 3 days post-surgery.

#### Medication

After the operation, the patients received a combination of ampicillin and sulbactam. Additionally, paracetamol is administered every 6 h and anti-inflammatory drugs are prescribed for excessive pain. Methylprednisolone was administered.

#### Oral and Dental Care

A chlorhexidine mouthwash was then applied.

#### Diet

The patients were advised to follow a clear liquid diet for the first 3 days, followed by a liquid diet for the next 3 days, and then transitioning to a soft diet for 7 days afterward.

#### Wound Care and Massage

Only iodine solution was used for scalp incision care during the first 2 weeks. After tape removal, lymphatic massage of the malar and temporal areas was recommended for 4 weeks.

### Statistics

IBM SPSS 27.0 package program (IBM Corp., Armonk, NY, USA) was used for statistical analyses. To assess the normal distribution of the data, Kurtosis and Skewness values were examined. Since the data showed normal distribution, paired samples test was used. A *p* value less than 0.05 was considered statistically significant.

## Results

**Fig. 7:**
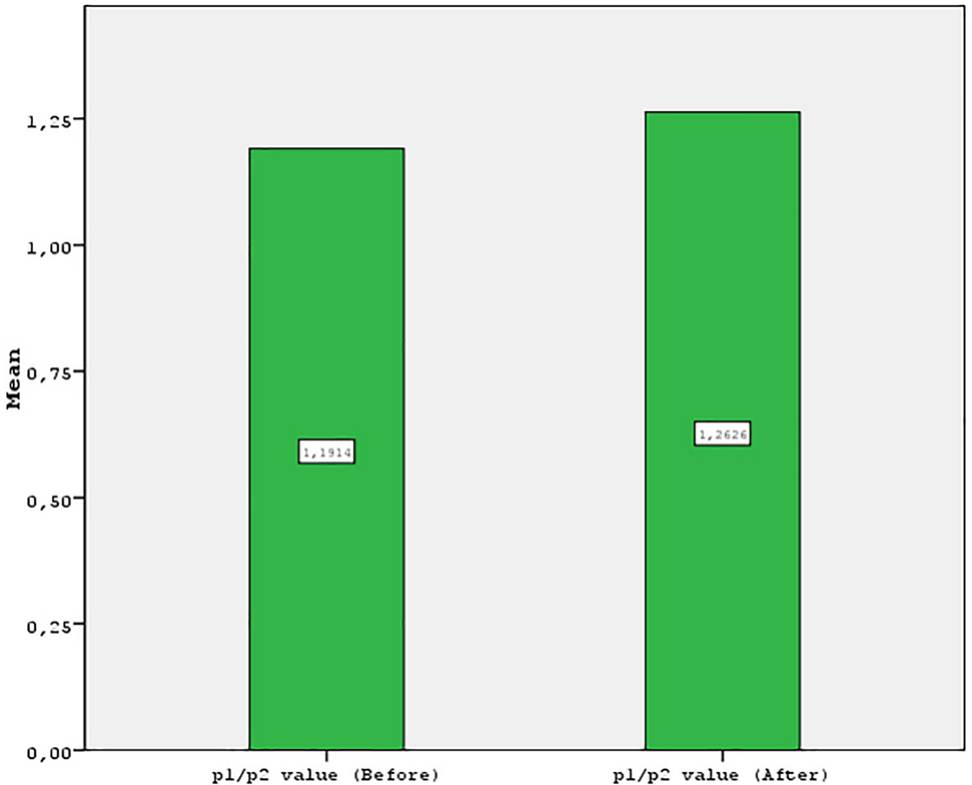
The graph shows P1/P2 rate results before and after

**Fig. 8:**
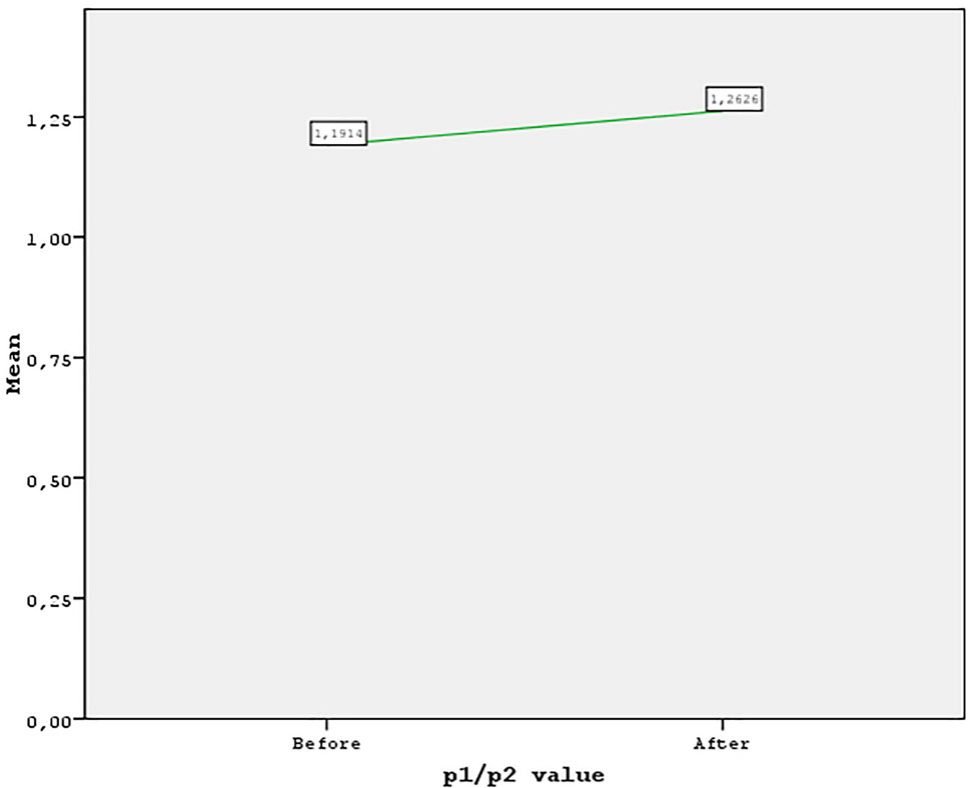
The graph shows P1/P2 rate results before and after

**Fig. 9:**
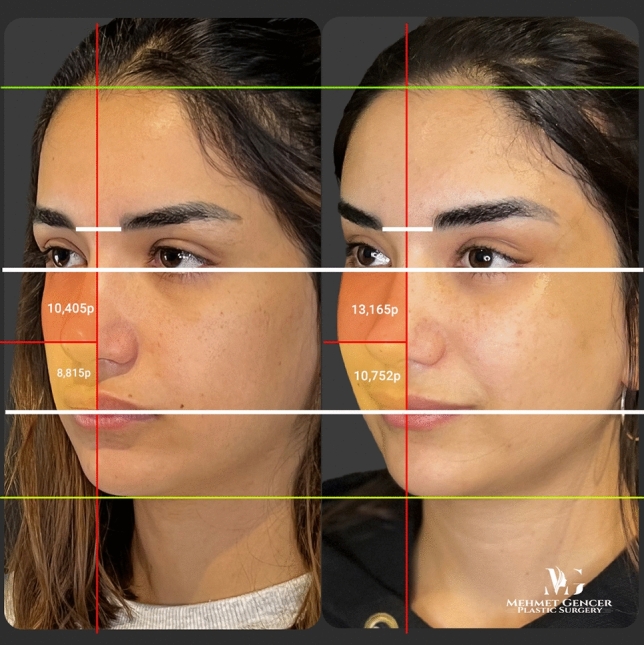
Preoperative and 1 year postoperative photographs of a 28-year-old female patient with P1-P2 calculation

**Fig. 10:**
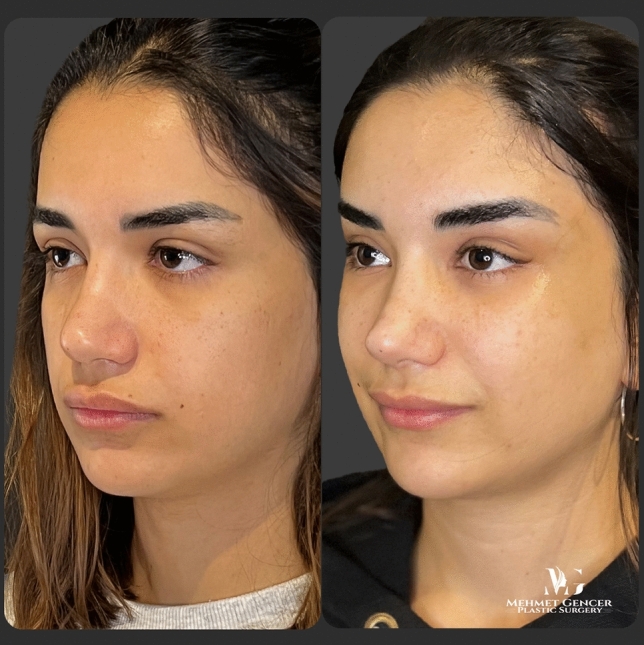
Preoperative and 1 year postoperative photographs of a 28-year-old female patient

**Fig. 11:**
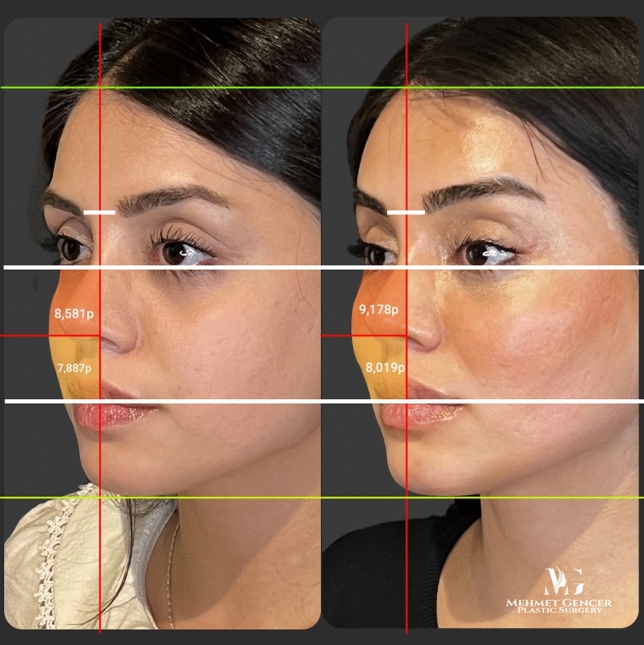
Preoperative and 1 year postoperative photographs of a 29-year-old female patient with P1-P2 calculation

**Fig. 12:**
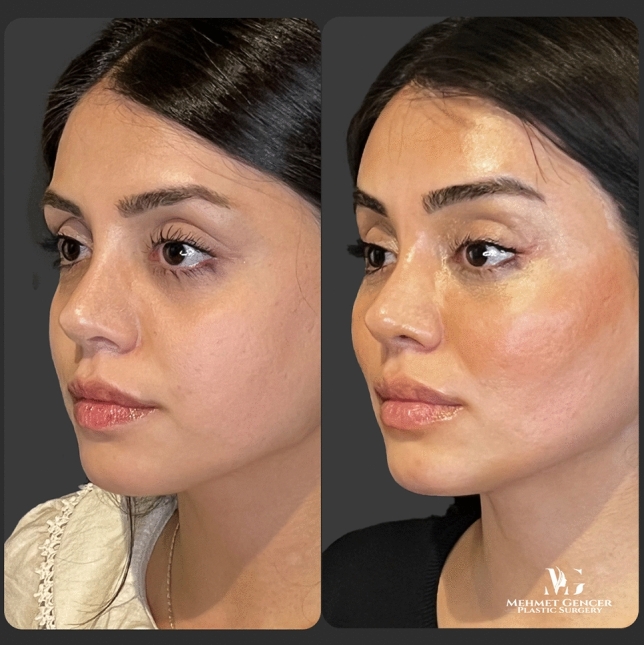
Preoperative and 1 year postoperative photographs of a 29-year-old female patient

**Fig. 13:**
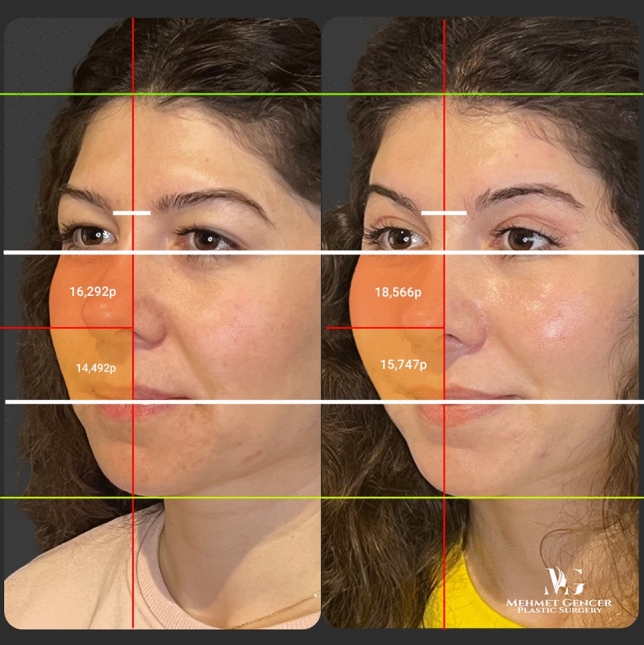
Preoperative and 1 year postoperative photographs of a 35-year-old female patient with P1-P2 calculation

**Fig. 14:**
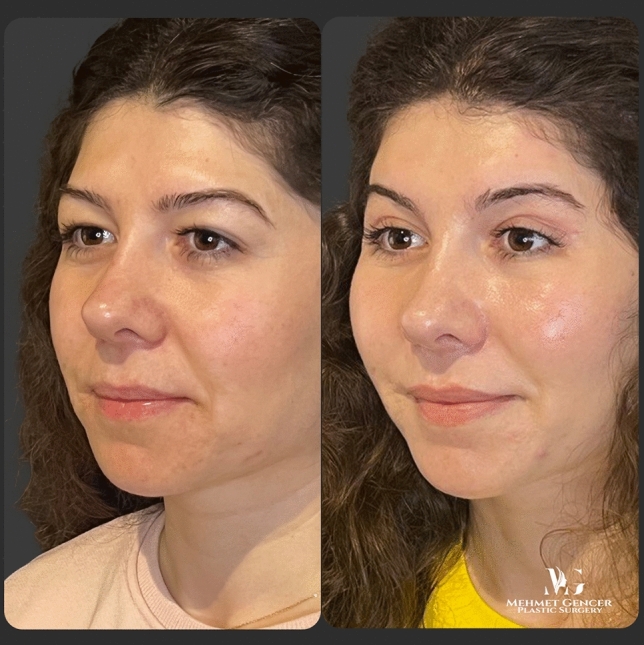
Preoperative and 1 year postoperative photographs of a 35-year-old female patient

**Fig. 15:**
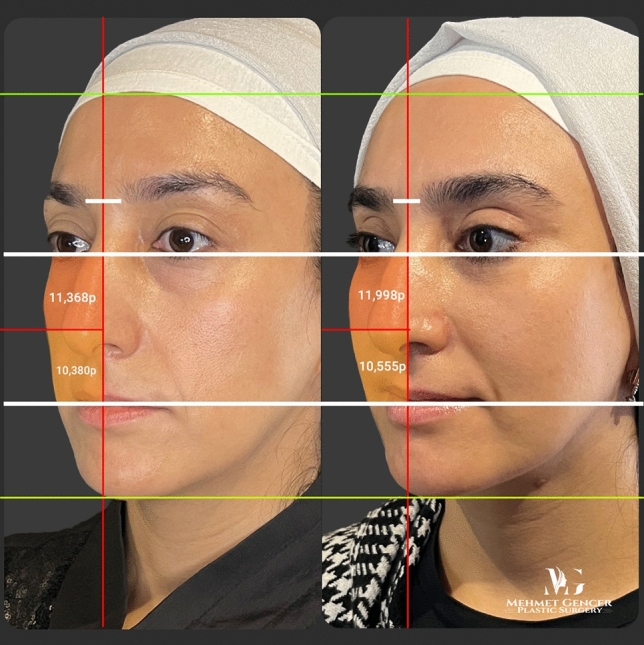
Preoperative and 1 year postoperative photographs of a 43-year-old female patient with P1-P2 calculation

**Fig. 16:**
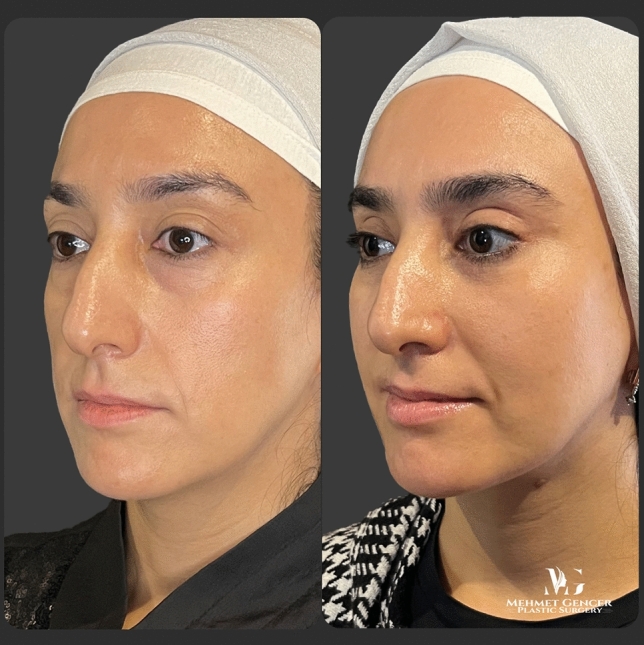
Preoperative and 1 year postoperative photographs of a 43-year-old female patient

This study included 184 patients with an average surgical duration of 130 min. The mean age of the patients was 31.4 years (range, 24–56 years). The mean follow-up period was 13 months (range, 12–19 months). Among the 184 patients enrolled in the study, infection occurred in five cases. The mean before the P1/P2 value score was 1.19 (± 0.14), and the mean after the P1/P2 value score was 1.26 (± 0.17). The difference between the before P1/P2 value and the after P1/P2 value scores was statistically significant (*p* < 0.05) (Figs. 7–16; Table 1).

**Table 1:** Postoperative outcomes and measurements

Information	Value	
Total number of patients	184	
Mean surgical duration (minutes)	130	
Mean follow-up period (months)	13	
Mean P1/P2 value before surgery (Mean±SD)	1.19 (± 0.14)	p < 0.05*
Mean P1/P2 value after surgery (Mean±SD)	1.26 (± 0.17)	
Infection	2.72%	

*SD* standard deviation

*Significantly different

### Complications and Their Management

#### Hypoesthesia in the Infraorbital Nerve Tract

An infraorbital sensory deficit is encountered in almost every case in the early postoperative period. Gentle traction and early administration of vitamin B supplements are the standard protocols for the management of hypoesthesia. Mean healing time of the sensory deficit was 72.4 days (0-272 days).

#### Bruising and Swelling

Observed in every patient. Head elevation and cold compression were applied for the first 3 days. Preoperative 1 mg/kg iv Methylprednisolone was administered in every case, and 0, 5 mg/kg was administered the next day.

#### Infection

Typical symptoms include pain and tenderness in the malar and periocular regions, fever, excessive edema, and erythema. Purulent discharge was observed. Streptococcus Gordinii was isolated from one discharge culture. Prophylactic antibiotic protocols were adjusted for penicillin-allergic patients by using clindamycin and moxifloxacin instead of ampicillin and amoxicillin. Upon suspicion of a clinical infection, the patient was readmitted, and treatment with clindamycin and linezolid commenced. All patients responded well and were discharged within 3 days but continued oral linezolid for an additional 10 days. Minor long-term asymmetry was noted in one case, while the remaining patients healed uneventfully. Important measures to prevent infectious complications include avoiding penicillin in trans-oral surgery, using water-tight mattress stitches for mucosal incisions, and performing frequent postoperative mouthwashes with chlorhexidine and/or iodine solutions.

#### Facial Nerve Neuropraxia

Buccal or zygomatic branch neuropraxia occurs due to traction or contact during suturing. It typically resolves within the first 3 weeks without any intervention.

#### Weakness in the Upper Lip Elevator Muscles

Weakness in the muscles may occur during subperiosteal dissection and adaptation and typically resolves by the first week without intervention.

## Discussion

As widely known, the term “midface” refers to the soft tissue area between the lower lid and the oral commissure. The anterior cheek structure in this area can be congenitally deflated or saggy in some individuals, or it can loosen due to the aging process [9–11]. This midfacial loosening and downward displacement of the anterior cheek results in a deeper nasojugal fold and nasolabial fold, which leads to the loss of youthful facial expressions [12, 13] Midface lift procedures aim to restore the youthful appearance and/or beautify the face in general, by lifting the downward displaced cheek/midface volume to a higher level on the anterior cheek.

The technique described in this study employs temporal incisions in the hair-bearing skin and intraoral incisions. These incisions are hidden and heal almost invisibly, especially in female patients. Temporal lifting procedures that use similar temporal incision and dissection patterns can easily be added to this operation. Compared to other face or midface lifting techniques (transpalpebral midface lift, lateral approach face lifts, etc.), this approach is almost completely free of scar-related complications, such as lower lid retraction, visible periauricular scars, loss of tragus, and earlobe deformities. As the frontal nerve is safely removed from the dissection zone, the most serious complication of face-lifting procedures, facial nerve injury, has a significantly reduced risk compared to the lateral approach techniques.

Owing to its anatomical nature, one cannot consider the midface as a separate structure from the lower lid, tear trough, and lateral canthus areas. Our technique, owing to the two-incision approach, provides perfect exposure and mobilization of the tear trough area along with the midface. In this way, midfacial volume shift can be achieved together with the correction of tear trough and beautification of the lid–cheek junction .^1,14,15^ Moreover, the lateral canthus can be freed as needed and its position can be adjusted accordingly, giving us the opportunity to adjust the canthal tilt without a subciliary incision and its consequences (lower lid retraction, ectropion, etc.). This makes our technique an effective treatment for patients with negative vectors, negative canthal tilt, and acquired lower-lid retraction problems.

The lift pattern of the midface in our technique was ectoral. The first suture, which fixes the midfacial flap to the orbital rim directly, provides a vertical vector lift, where the soft tissue is pulled directly to the rim. As this is not a cable suture, it enables direct contact of the periosteum on the flap base with the rim bone with a direct pull force. This vertical lift recruits a small volume to the lower rim. The rest of the midface suspension sutures pulled the midface flap in the superolateral direction, which was the second vector.

Both deep and superficial plane lifts have advantages. When one aims to mobilize and redistribute the skin, superficial plane dissections are more effective, as they allow more mobility. However, with superficial plane dissection, volume shift or recruitment is limited. A deeper dissection plane should be used to obtain a thicker flap that can carry more volume to the target area. In our technique, which is described as “dual-plane,” we aim to take advantage of both deep and superficial plane dissections. The anterior maxilla was dissected in the subperiosteal plane, which allowed a sufficient volume shift to the upper midface. In the prezygomatic space, dissection is performed at two different levels: the sub-SOOF and the subperiosteal. Superiosteal dissection enables an effective volume increase over the lower rim, while the sub-SOOF dissection redrapes the orbicularis and skin, softening the transition of volumetric change in the midface.

The technique described here has some drawbacks to infection. It has been observed that creating a hole in the inferior rim fundamentally leads to two problems. First, because the vector of the lift is vertical, an increase in intraocular pressure is observed in most patients, leading to chemosis. In addition, the tip of the device can break while drilling the bone. In five of our presented cases, the motor tip broke, and the remaining fragments were removed by shaving. None of the patients had any fractures. Moreover, our technique involved the use of multiple sutures. Fixation of the origin of the temporal muscle can result in palpable knots from the outside, especially in patients with thin skin, and can sometimes cause small granuloma formation. In such cases, patients can be treated with epithelializing creams without the need for intervention.

Of course, this technique has some limitations and drawbacks. Patients with heavy faces and prominent cheekbones are not good candidates for this procedure. In addition, patients with severe lower face laxity/jowling should be treated using lateral approach techniques instead of the described technique. The greatest disadvantage of our technique is the relatively high risk of infection. As our experience shows that all infected patients were allergic to penicillin, this can be considered an important criterion for patient selection.

This article describes a dual-plane (sub-SOOF and sub-periosteal) bi-vectoral (vertical and superolateral) midface lift performed using transtemporal and transoral approaches. This technique can be considered an effective method of midfacial lifting for beautification or rejuvenation.
